# Standard reporting of high-risk histopathology features in retinoblastoma

**Published:** 2018-06-03

**Authors:** Caroline Thaung, Esin Kotiloglu Karaa

**Affiliations:** 1 Consultant Ophthalmic Pathologist: Moorfields Eye Hospital, Institute of Ophthalmology, London, UK. *Teaching blog:* **https://eyepathlondon.wordpress.com/**; 2 Consultant in Paediatric Pathology: Barts Health NHS Trust, London, UK.

**Once an eye with retinoblastoma is excised, accurate histopathological staging is essential in order to determine whether the child can leave the hospital completely cured, or may need chemotherapy or radiotherapy.**

**Figure 1:**
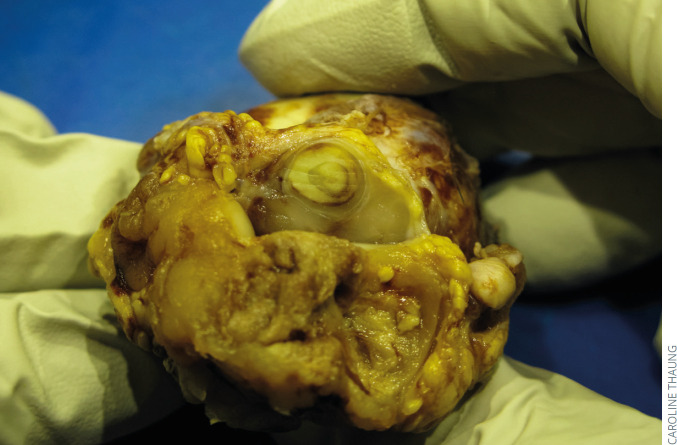
If there is obvious extraocular extension such as an extrascleral nodule or orbital involvement in an exenteration specimen, the specimen should be sampled in such a way as to confirm this microscopically.

Surgical treatment for retinoblastoma is limited to enucleation (removal of the eye) or exenteration (removal of the eye and contents of the orbit). Histopathological examination of such specimens yields important information which must be taken into account when considering prognosis and making decisions about future management and treatment.

## Standard histopathological reporting

When histopathologists are dealing with cancer specimens, their reports should include standard and appropriate information items, commonly known as a dataset. In the UK, histopathologists use datasets produced by the Royal College of Pathologists. Data items are reviewed every two years and aligned with the international tumour, node, metastasis, (TNM) classification. The TNM classification for retinoblastoma has recently been updated from TNM7 to TNM8. The newest dataset for retinoblastoma from the Royal College of Pathologists (RCPath), released in early 2018, reflects the changes in the TNM classification. Visit **http://bit.ly/RBdataset**

### Histological high-risk features: rationale

The RCPath dataset for retinoblastoma records whether tumour is present in specific structures. Presence of tumour in any of these structures is considered a histological high-risk feature (HHRF). Retinoblastoma specimens with HHRFs indicate that the patient is at higher risk for metastasis (tumour spread) than a patient whose specimen does not have such features.

Detection of HHRFs by the histopathologist influences management decisions. The histopathologist examining the specimen should therefore specifically seek such features. The structures where tumour presence is regarded as an HHRF are:

- anterior chamber
- iris
- trabecular meshwork
- Schlemm's canal
- ciliary body
- choroid (above a certain threshold)
- sclera
- extraocular structures
- retrolaminar optic nerve (including the cut end).

### Gross examination of specimens

The RCPath dataset for retinoblastoma includes recommendations for macroscopic examination and sampling of globe and exenteration specimens. Even if resources are limited, it is useful to sample the cut end of the optic nerve separately (if length allows) as well as a ‘PO block’ of the globe (see panel). If the globe is being opened fresh to sample tumour for cytogenetics, the optic nerve should be sampled first. This will avoid artefactual contamination.

The sampled optic nerve should be embedded transversely, so that a full cross-section can be evaluated. It can either be embedded on the true resection margin (surgeon's cut end) or the pathologist's cut end, as long as the orientation is known to the reporting pathologist.

For further information on what to look for microscopically (with figures), read this article on **www.cehjournal.org**.

**PO block**

This is a slice which includes the cornea, pupil and optic disc. It is typically obtained by making two parallel slices from anterior to posterior, one either side of the limbus. The orientation of the parallel slides may be sagittal, transverse or oblique, depending on the location of the tumour and any other pathology to be sampled.
